# A quasi-experimental study of impacts of Tanzania’s wildlife management areas on rural livelihoods and wealth

**DOI:** 10.1038/sdata.2018.87

**Published:** 2018-07-03

**Authors:** Jevgeniy Bluwstein, Katherine Homewood, Jens Friis Lund, Martin Reinhardt Nielsen, Neil Burgess, Maurus Msuha, Joseph Olila, Sironka Stephen Sankeni, Supuku Kiroiya Millia, Hudson Laizer, Filemon Elisante, Aidan Keane

**Affiliations:** 1 Department of Food and Resource Economics, University of Copenhagen, Rolighedsvej 25, 1958- Frederiksberg, Denmark; 2 Department of Anthropology, University College London, 14 Taviton St, London WC1H 0BW, UK; 3 UNEP—WCMC, Cambridge CB3 0DL, UK; 4 Ngorongoro Conservation Area Authority, Community Development and Tourism, P.O. Box 1, Arusha, Tanzania; 5 Tanzania Natural Resource Forum, P.O. Box 15605, Arusha, Tanzania; 6 Environment and Society Program, 233 UCB, University of Colorado, Boulder, USA; 7 Ujamaa Community Resource Team (UCRT), P.O. Box 15111, Arusha, Tanzania; 8 Department of Natural Sciences, Mbeya University of Science and Technology, PO Box 131, Mbeya, Tanzania; 9 Department of Conservation Biology, School of Biological Sciences, University of Dodoma, P.O. Box 259, Dodoma, Tanzania; 10 School of GeoSciences, University of Edinburgh, EH9 3FF, Edinburgh, UK

**Keywords:** Socioeconomic scenarios, Sustainability, Developing world, Agriculture, Interdisciplinary studies

## Abstract

Since the 2000s, Tanzania’s natural resource management policy has emphasised Wildlife Management Areas (WMAs), designed to promote wildlife and biodiversity conservation, poverty alleviation and rural development. We carried out a quasi-experimental impact evaluation of social impacts of WMAs, collecting data from 24 villages participating in 6 different WMAs across two geographical regions, and 18 statistically matched control villages. Across these 42 villages, we collected participatory wealth ranking data for 13,578 households. Using this as our sampling frame, we conducted questionnaire surveys with a stratified sample of 1,924 household heads and 945 household heads’ wives. All data were collected in 2014/15, with a subset of questions devoted to respondents’ recall on conditions that existed in 2007, when first WMAs became operational. Questions addressed household demographics, land and livestock assets, resource use, income-generating activities and portfolios, participation in natural resource management decision-making, benefits and costs of conservation. Datasets permit research on livelihood and wealth trajectories, and social impacts, costs and benefits of conservation interventions in the context of community-based natural resource management.

## Background & Summary

Community-based conservation and natural resource management interventions (CBC, CBNRM) promise both biodiversity conservation and rural development. Conservation interventions may be more socially and ecologically sustainable, if local people benefit^1^. Yet the relationship between conservation and development remains inconclusive^2^, and social impacts of conservation are commonly unevenly distributed in terms of geography, ethnicity, class and gender^3^.

Literature calls for rigorous impact evaluation design to assess social impacts of conservation interventions^4,5^. Responding to these calls, we conducted a quasi-experimental impact evaluation of Tanzania’s community-based wildlife conservation policy. This policy envisions direct engagement of local people in wildlife conservation on village lands through implementation of Wildlife Management Areas (WMAs). A WMA spans several villages adjacent to protected areas. Villages set aside significant parts of village land as WMA in return for benefits derived from external wildlife/nature-based tourism investments on that land. Village assemblies elect local representatives to the Community-Based Organization (CBO) charged with managing a WMA. Village game scouts (VGS) are hired to patrol WMAs against forbidden activities (e.g., trespassing, agriculture, livestock grazing, hunting and collection of timber and non-timber forest products). This creates conditions whereby some groups/individuals stand to benefit from WMAs while others may be made worse off.

The datasets reported here formed part of PIMA, the first large-scale study designed to evaluate impacts on rural livelihoods of WMA policy implementation since mid-2000s. PIMA stands for Poverty and Ecosystem Impacts of Tanzania’s Wildlife Management Areas, and was funded by the Ecosystem Services and Poverty Alleviation (ESPA) programme. Data were collected during surveys conducted with households from WMA-member villages and households in a statistically matched sample of villages that were not part of a WMA at any time. The surveys asked about both contemporary conditions and recall of conditions at a 2007 baseline, when first WMAs became operational. Household questionnaires encompass household demographics and assets, household head’s gender and social status, land and livestock ownership, material benefits from tourism, human-wildlife conflict, wealth trajectories, income-generating activities, environmental resource dependency, and involvement in local decision-making about natural resource management. Wives’ questionnaires enabled a gendered analysis of social impacts of WMAs and focused on women’s self-reported perceptions of access to land, resources, income-generating activities, human-wildlife conflict, participation in WMA management, autonomy, remittances, and perceived costs and benefits of membership in WMA villages.

**Figure 1:**
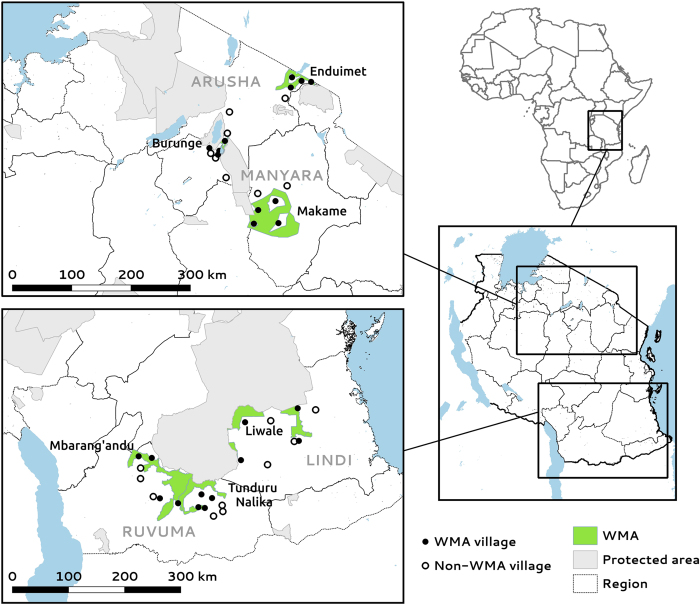
Map of study villages in regional context and in relation to WMAs and protected areas.

This study generated three livelihoods datasets. We first collected participatory wealth ranking data for 13,578 households, representing all registered households that had resided since at least 2007 in 42 villages. Of these, 24 villages were members of six different WMAs across two geographical regions, and 18 were statistically matched non-WMA villages (Fig. 1, Table 1). Non-WMA villages were matched to WMA villages using nearest-neighbour matching with replacement based on Mahalanobis distance, calculated from relevant environmental, conservation and socio-economic covariates measured prior to WMA implementation (Table 2). In each village, focus groups established four different wealth strata to rank all households as Very Poor, Poor, Normal or Rich. This large-scale wealth ranking was analysed in its own right and used as the sampling frame for a stratified random sample of 1,924 household heads and 945 household heads’ wives who completed a detailed questionnaire survey.

The six sampled WMAs represented one-third of the 18 WMAs implemented in Tanzania at the time of the study (2014/15). WMAs were purposively selected to include some of the earliest WMAs established (to allow sufficient time for an impact to be observed), and to represent variation in ecosystem type and tourism potential^6^ (Table 1). Only WMAs with at least four member villages were considered. WMAs where pre-WMA socio-economic data were available (to facilitate validation of recall) were preferred. This sampling strategy enables spatially and agroecologically disaggregated analysis of potential effects of conservation and tourism on rural livelihoods.

Future uses of datasets may include research on Tanzania’s rapid socio-economic change and growing inequalities^7^ using data on *change* in wealth (dataset 1), land ownership, cultivation, and livestock assets (dataset 2), along class and gender lines (datasets 2 and 3). These datasets could also be used alongside environmental data collected from the same areas and at a similar temporal scale to allow combined socio-ecological impact evaluation. Published and forthcoming papers from this research provide context, qualitative analysis, technical background and test theories of change to better situate future analyses of these datasets^8–15^.

## Methods

### WMA village sampling and statistical matching of non-WMA villages

Within each of six WMAs we first chose four villages per WMA as follows. Where one of the WMA villages hosted the CBO (Community-based organisation), this village was included in the sample. Three additional villages were randomly sampled from the remaining WMA member villages. Where the CBO office was located outside of the WMA villages, all four villages were randomly sampled (Table 3). In Burunge WMA, we omitted one of the villages from consideration (Minjingu). Minjingu has rejected being part of the WMA since Burunge’s registration and has refused any money from tourism income ever since.

We used country-wide information from the 2002 national census on boundaries of enumeration areas (EAs) as our sampling frame to match WMA and non-WMA villages. Enumeration areas are administrative units defined for the purposes of conducting censuses which each contain a certain number of households. In rural areas they usually correspond to a single village, collection of smaller villages or part of a larger village. EAs were used in place of villages as the unit of sampling and matching because equivalent information was not available at the village level. In Southern WMAs, EAs poorly represented some of the village boundaries on the ground. In the case of Mbarang’andu WMA we were able to obtain locally more accurate maps covering the whole of Namtumbo district which were georeferenced and used to correct village boundaries prior to matching. For Tunduru Nalika WMA, equivalent maps covering all of Tunduru district were not available so the census EA boundaries were used despite discrepancies between 2002 shapefiles and information on the ground.

To select non-WMA villages that were as similar as possible to our sampled WMA villages prior to WMA establishment, we pursued a matching approach drawing upon existing available georeferenced data covering Tanzania relating to the period before WMA establishment (Table 2). Non-WMA villages were selected using nearest neighbour matching with replacement based on Mahalanobis distance metrics calculated from a set of economic, environmental and demographic variables. These variables were chosen because they were theorised to affect both the probability that a village would become part of a WMA and the effect of a village joining a WMA. The pool of potential matches was restricted to villages within the same region of Tanzania. Highly skewed variables were transformed prior to use in matching. The matching procedure was carried out in R using the Match function from the Matching package^16^.

### Constructing a village register

Within each village, we sampled households from different wealth-based strata. To identify eligible households and to rank each household according to wealth we first needed a village register for each village. The following eligibility conditions were applied to construct the village register:

- We assumed patriarchal intra-household dynamics. A household constitutes multiple sub-households in case of polygamous marriages where the husband is in charge of economic decision-making on behalf of other family members. Hence the male head of the polygamous family represents the entire household and potential sub-households. Sub-households are not recorded in the village register to avoid double counting. This differs from the approach used by The Nature Conservancy in a subsequent survey^17^.
- A son becomes a head of his own household as soon as he is making his own decisions over his family’s economic activities. In this case he is treated as a head of the household and included in the sample frame.
- Spatially, several economically independent households and by extension several household heads can share a multi-household homestead and each of these were eligible.
- Households had to be formed at least in 2007 or earlier to be included.
- Households that had immigrated to this village, had to be present in this village at least from 2007 onwards to be included.
- The head of the household can be female in cases where the head is a widow or a never married woman, where the husband left or was permanently not available, the husband is disabled, or the husband is alive but does not provide for the household.

The rationale for exclusion of households that were not formed prior to 2007 or immigrated into the village after 2007 is that the survey seeks to understand what has happened to the household from 2007 until the time of the survey (2014/15). See below for the method of pinpointing recall.

Following these eligibility criteria, a village register was obtained from the village office, screened and updated. If no village register was available, it was constructed with the help of knowledgeable people, usually village and subvillage chairmen and elders. Most of the time these people were men. A paper-based template was used, and later transcribed in Microsoft Excel.

### Event calendar for recall data

We used recall of circumstances in 2007 prior to implementation of the relevant WMAs and included description of a prominent event as baseline to facilitate recollection. In the North, respondents were asked to recall the September 2007 eruption of Ol Doinyo Lengai volcano (mountain sacred to Maasai), as its effects were widely felt across the region, affecting crop and livestock production both directly and then throughout the year that followed. In the South, where the eruption was less salient, people were asked to think back to President Kikwete’s 2005 election as a help to remember 2007 events. In both cases people’s recall was further prompted by reference to local events (local elections; village subdivisions) and memorable personal or family matters (birth of a child, construction of a house…). By focusing on important and easily quantifiable household assets we reduced potential implications of recall bias.

### Participatory wealth ranking

After the village register was constructed, wealth ranking was conducted with the purpose of assessing the wealth trajectory of all eligible households in the village register in terms of development in relative wealth over time, based on four community-distinguished and community-defined, levels of wealth.

Focus groups were predominantly male. Prior to asking a focus group to rank households from the updated PIMA village register, we conducted a focus group discussion on what constitutes material wealth in each village individually. We asked the group to discuss together what constitutes a state of poverty in the eyes of the community and when a household might be considered very poor, poor, normal, and rich. Typically, we asked the focus group participants questions like *what would a typical very poor/poor/normal/rich household in your village..*

- spend money on, spend additional money on if it becomes available,
- be able to afford in terms of access to food
- have as ability to send children to school, to pay back debt, to be able to repair the house
- be able to do to improve agricultural land or to increase livestock herd size, or to acquire more land
- have as measures of housing quality including iron sheet roof, cement floor, electricity etc.
- have as assets such as clothes, radio, mobile phones, furniture, bicycles, domestic animals, farm land, agricultural implements, bee hives, small scale businesses, shop etc.
- have in terms of access to services such as water pump, health care, veterinary assistance, primary and secondary school for children, number of meals per day etc.
- have in terms of access to opportunities such as non-farm employment, pension, micro loans, bursaries etc.

The goal was to identify four distinct wealth groups within the community. The discussion was led by PIMA research assistants (enumerators, co-authors to this article) who would prompt the focus group participants if the discussion did not yield sufficient differentiation between the different wealth categories. After the four wealth groups had been identified by as much consensus as possible, the research assistants wrote down the defined assets and associated quantities for each wealth rank category. Based on this shared understanding of what constitutes poverty and wealth in the village, the focus group would together proceed to determine for each of the households within the PIMA village register its present-day (2014/2015) and 2007 wealth rank as *Very poor, Poor, Normal* or *Rich*. The focus group assessed each household as compared to the village in general.

The results were written down by the research assistants into the village register template and transcribed into Microsoft Excel. This yielded the first dataset, including 13,578 households across 42 villages (villages, as defined by the 2002 census, PIMA_WEALTH_RANKING.csv, Data Citation 1).

### Stratified random sampling

After a complete village register was created based on the PIMA eligibility criteria, village leaders residing in the same village were identified following a set of criteria (‘leadership positions’):

- WMA CBO member, member of WMA board of trustees (typically 3-4 per WMA village)
- WMA Village Game Scout-VGS (typically 3-6 per WMA village)
- Village Chairman-VC (1 per village)
- Village Executive Officer-VEO (0-1 per village)
- Ward Executive Officer-WEO (0-1)
- Subvillage Chairman-subVC (typically 3-4 per village)
- Chair, secretary or treasurer of Village Natural Resource or Environmental Committee (typically 1-3 per village)

Thus, leaders in categories A and B were only present in WMA villages. In addition to wealth ranking, the identification of households with members in village leadership positions enables the analysis of social impacts on rural livelihoods to be differentiated between households whose members occupy leadership positions and those which do not. Both wealth ranking data and village leadership data were used for stratified random sampling.

In each village 40 households were randomly sampled for inclusion in the main household questionnaire survey, which was conducted with the household head (PIMA_HHHEAD_SURVEY.csv, Data Citation 1). To ensure that different wealth categories and social strata were sufficiently well represented to allow differentiated statistical analysis, the household stratification and sampling procedure was designed to oversample poor households and leaders, selecting randomly:

- 10 ‘very poor’ households (according to wealth ranking as of 2007)
- 10 households with household members in ‘leadership’ positions (as of 2014/15)
- 20 households from the rest of the village register (the ‘other’ stratum).

In WMA villages the 10 leadership households were identified based on the ‘leadership positions’ in the village register. Given that many control villages did not have 10 ‘leadership’ households as defined above, ‘rich’ households based on wealth rank in 2007 were added to attain 10 households in this stratum.

Alongside the main household survey, we also randomly sampled 20 wives of male heads of sampled households to conduct a household survey specifically targeting wives of male household heads (PIMA_WIFE_SURVEY.csv, Data Citation 1). The relative proportions of wives sampled within the three strata were the same as those of household heads in the main survey. If the head of a selected household or the wife were not available, could not recall their situation in 2007 with clarity, or refused to participate, a backup respondent was selected (see Table 4). This was done by selecting the first respondent on the backup list (not the one geographically nearest to the enumerator’s position in the village).

Since the 2002 census some of the villages selected for inclusion in our study have split into new villages. To deal with this we drew our household sample from within the original village boundaries. Thus, we created sample frames (across several new villages) for the 42 original villages selected (although 5 additional villages had been created since 2002), and selected 40 households from within each of these original villages. That way the household numbers remain constant in cases where original villages split into several new ones.

### Survey implementation

Both household survey questionnaires were initially co-designed by University of Copenhagen and University College London researchers, and subsequently trialled and refined in discussion with local research assistants. The survey questionnaires were produced in Microsoft Word and subsequently digitised using OpenDataKit (ODK), an open source survey package that runs on Google Android smartphones and tablets through the app ODK Collect. ODK stores both the questionnaire and survey results on a secure, password protected webserver (ODK Aggregate).

Four experienced research assistants (RAs) were recruited by the first author with the assistance of the local project partner Tanzania Natural Resource Forum (TNRF), and trained as enumerators by the first author in the field. Each enumerator received a tablet for data collection purposes, and was only required to use paper for additional opportunistic field notes, or in case of technical failure. Household survey questionnaires were pre-tested in the field under the supervision of the first author as field team leader and instructor. Training included a week of theory on socio-economic development and change, and role-play in conducting household surveys, and two weeks of hands-on training by conducting surveys using paper and ODK tablets in the villages. Enumerators individually recorded questionnaire results from the same, jointly conducted, household survey in the presence of the first author. These individual results were compared against each other to establish a common understanding of the meaning and purpose of individual survey questions. All enumerators were male and fluent in both Kiswahili and English. Two of the four enumerators were also fluent in Maa, which was important in some of the villages in Northern Tanzania. Given the range of local languages used in the study areas, questionnaires were not translated into Swahili or Maa. Instead, the first author worked with the field teams to ensure that RAs had a robust and consistent individual and collective understanding of the meaning and purpose of each question, and that each could be presented unambiguously to interviewees irrespective of the operational language.

The first author supervised the field team for six out of 12 months of data collection, and conducted regular quality control remotely through ODK’s data server for the entire period of data collection. During the absence of the first author, other authors individually spent additional periods of a week or so at a time with either the northern or the southern teams to observe data collection procedures and techniques, and help resolve emergent issues.

The survey was approved by the UCL Anthropology Departmental Ethics Committee under the reference No. Z6364106 of UCL Data Protection Registration. The survey obtained a research permit (2014-49-NA-2013-154) from Tanzania Commission for Science and Technology (COSTECH). All survey respondents were introduced to the project using a plain language statement, which explained the collaboration and project aims and methods, and included information about the interviewees’ right to drop out, prior to or at any time during the survey. Respondents were then asked for their informed consent to participate in the survey. Participation was voluntary and no cash payment was offered. Small inexpensive but locally appreciated courtesy gifts such as tea, sugar or phone credit vouchers were offered as a thank you after the survey.

Both questionnaire surveys and wealth ranking took place between May 2014 and May 2015.

### Questionnaires

Household head and wives’ questionnaires (PIMA_HHHEAD_SURVEY_INSTRUMENT.pdf, PIMA_WIFE_SURVEY_INSTRUMENT.pdf) are provided in pdf form and correspond to datasets 2 and 3 (PIMA_HHHEAD_SURVEY.csv, PIMA_WIFE_SURVEY.csv). The questionnaires are structured as follows in Table 5. Each question is numbered accordingly and corresponds to the header in the datasets. E.g., question 6.2 in pdf file of questionnaire with household head corresponds to the column with the header ‘q6.2’ in the csv file of dataset 2. Some questions could be answered with the ‚other‘ option, and were pre-coded as such. In this case the enumerator recorded the answer as open text. E.g., question 6.2 in pdf file of questionnaire with household head can be answered with ‘other’ under code number 7. The corresponding column in the csv file containing this response is named as ‘q6.2_other’.

Most questions were pre-coded. Questions 3.1 and 11.7 in questionnaire with household head, and question 8.8 in questionnaire with wife of household head were open text. Sections that were only covered in WMA villages are indicated with * (see Table 5). Many questions aim to elicit if there has been a change in a particular condition as perceived by the respondent (e.g., change in access to water or firewood), and what was the main reason for change according to the respondent (e.g., access became restricted due to..). For that purpose, a question was asked both pertaining to the situation in 2007 (recall baseline) and to the present (2014/2015). In case there was change, and in case the survey was conducted in a WMA village, the enumerator would record if the perceived change was perceived to be due to the implementation of the WMA. However, the enumerator would not prompt about the WMA specifically, when asking about reasons for change, to avoid strategic responses.

### Scoring exercise

Section 10 of questionnaire with household head was designed as a scoring exercise to establish the relative importance of different components of household livelihoods and income portfolios, in the six categories agricultural production, livestock production, environmental product harvesting, wage employment, business, and remittance and pensions. Similarly, section 9 of questionnaire with household head focused specifically on the relative importance of nine different categories of environmental goods and services including firewood, charcoal, construction material, timber, fish, honey, bushmeat, wild vegetables and fruits, and the category, other, containing all other environmental products. Participants were asked by the enumerator to distribute a pre-defined amount of maize grains (50 and 25 grains in section 9 and 10, respectively) across the different categories to reflect the importance in the household’s livelihood during the past 12 months at the time of the enumeration in 2014/2015. Importance was explained as encompassing both cash income and value for own subsistence use defined as replacement value. Printed templates in Kiswahili were used with appropriate pictures to facilitate the exercise. After grains were placed and the enumerator had recorded their distribution, the respondent was asked to think back to 2007 and move the grains around accordingly to reflect relative changes across categories. This also included removing or adding as much extra maize as necessary to reflect changes in overall household income summed across the items listed, in cash and in kind, between 2007 and 2014/2015.

## Data Records

Three different datasets are available (Table 6):

- Village-based wealth ranking (n=13,578, Villages and households are anonymised, PIMA_WEALTH_RANKING.csv, Data Citation 1).
- Socio-economic household survey with the head of the household (n=1,924, Villages and households are anonymised, PIMA_HHHEAD_SURVEY.csv, Data Citation 1).
- Socio-economic household survey with the wife of the household head (in case the head of the household is male, n=945, Villages and households are anonymised, PIMA_WIFE_SURVEY.csv, Data Citation 1).

The survey data is provided as comma-separated value (csv) files. All variables in the csv format correspond to the number in the questionnaire. Both household survey questionnaires can be downloaded in English as pdf files, including full code lists.

## Technical Validation

**Figure 2:**
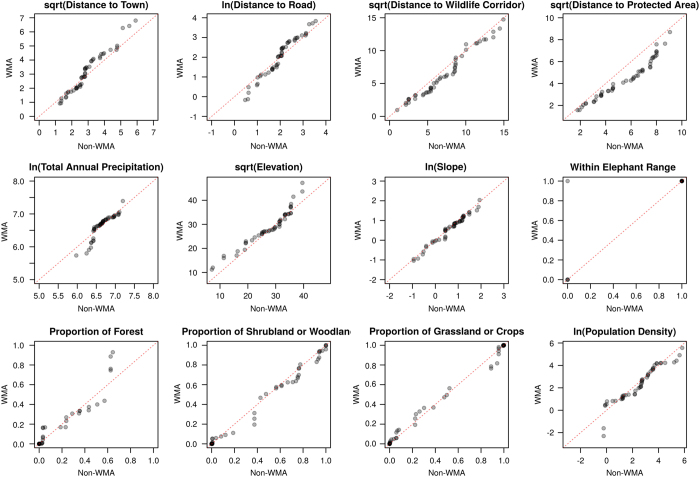
QQ-plots comparing the values of each of the matching covariates in the matched pairs of WMA and non-WMA villages included in the study.

The quality of the match between selected WMA and non-WMA villages was assessed by examining the improvement in covariate balance achieved after matching via standardised mean differences (Table 7). Balance improved for all covariates, but a non-negligible level of imbalance remained in the distance from protected areas, the distance from wildlife corridors and population density due to the limited availability of non-WMA villages with similar characteristics to WMA villages. We also visually examined QQ-plots of the matched pairs of WMA and non-WMA villages to determine specific areas where differences in observed covariates remained after matching (Fig. 2).

The quality of wealth ranking is ensured by pre-testing the approach in four villages where each village was ranked by two different focus groups to compare the results. We conducted wealth ranking with a female and a male focus group separately, each comprising 2-4 people knowledgeable of village affairs and households. Often the 2-3 people in the focus group would be sub-village chairpersons. Generating two separate wealth rank datasets (with a male and a female focus group) proved time consuming. After an analysis of difference indicated that the two sets were not very different, it was decided to proceed with only one focus group in each village.

**Figure 3:**
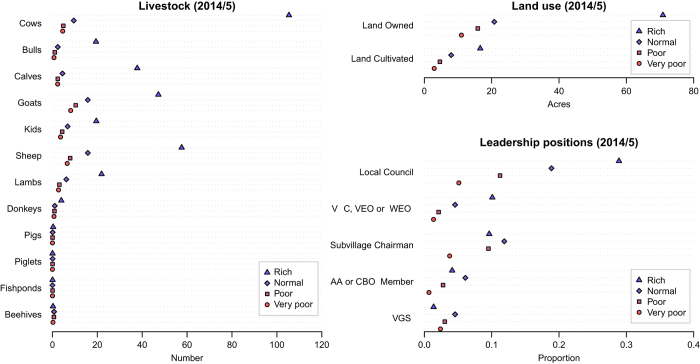
Mean number of livestock owned, acres of land used and proportion with members who occupy a leadership amongst households grouped by wealth rank in 2014/5.

After the main household survey data had been collected, we also used this to verify that the wealth ranks had correctly differentiated between households possessing differing amounts of land and livestock and households which were more likely to have members who occupy positions of leadership (Fig. 3). Our *a priori* expectation was that higher wealth ranks would be associated with ownership of more assets and a higher probability of occupying a leadership position.

Household survey questionnaires reflected project research questions and objectives and were designed collaboratively, drawing on the extensive expertise of different research project partners pertaining to socio-economic livelihood and anthropological studies. An important criterion was to make sure that a questionnaire could be conducted within 1-1.5 h to avoid respondent fatigue and thereby ensure high quality data, that respondent’s needs were respected and that any collection of data not of immediate relevance to the research project objectives was avoided.

ODK enabled a digital data entry that proved to save time, reduce errors and offered almost real-time data availability for remote quality control. Paper was only used during training in combination with ODK tablets, and as a backup in case of technical failure of tablets (which did not happen). Typographic errors and other forms of human error were minimized substantially by the inclusion of data validation rules in the ODK code for the questionnaire surveys, implemented by the first author. For example, string answers were not accepted where a number (integer) is expected, logical upper and lower numerical limits were pre-coded to reduce typographic errors; skipping and branching logic ensured that only appropriate questions were asked; questions that were required to be asked were pre-coded appropriately.

Due to the inability to make online corrections to the ODK dataset, the first author documented all inaccuracies and errors in a log spreadsheet throughout the 12 months of data collection. This was used to clean the complete dataset after data collection finished.

## Usage Notes

To account for the residual imbalance in observable covariate values that remained between WMA and non-WMA villages after matching, we suggest that future analyses of the data should include adjustment on the matching covariates as part of their modelling strategy. Where appropriate, analyses of data from the household head survey and wife survey should also be designed to account for the stratified sampling approach adopted during data collection (e.g., using sampling weights or post-stratification based on the village wealth ranking data which served as our sampling frame).

A range of manuscripts is currently being developed based on analysis of various aspects of the PIMA data which we aim to publish in international peer reviewed scientific journals. Hence, anyone wishing to use the same specific data should consult the corresponding author of these publications in preparation. This includes the following data/questions:

Martin R. Nielsen (mrni@ifro.ku.dk): Sections 9 and 10 of the questionnaire conducted with the household heads (Dataset 2, PIMA_HHHEAD_SURVEY.csv).

Aidan Keane (aidan.keane@ed.ac.uk): Evaluating socio-economic effects of conservation and development interventions through participatory wealth change analysis (Dataset 1, PIMA_WEALTH_RANKING.csv).

Jevgeniy Bluwstein (j.bluwstein@gmx.de): Livelihoods impacts of community-based conservation (Dataset 2, PIMA_HHHEAD_SURVEY.csv)

Katherine Homewood (k.homewood@ucl.ac.uk): Women, well-being and community-based conservation (Dataset 3, PIMA_WIFE_SURVEY.csv).

## Additional information

**How to cite this article:** Bluwstein, J. *et al.* A quasi-experimental study of impacts of Tanzania’s wildlife management areas on rural livelihoods and wealth. *Sci. Data* 5:180087 doi: 10.1087/sdata.2018.87 (2018).

**Publisher’s note:** Springer Nature remains neutral with regard to jurisdictional claims in published maps and institutional affiliations.

## Supplementary Material



## Figures and Tables

**Table 1:** WMA sites.

**Ecosystem type**	**WMA**	Tourism potential and type*	**Rural livelihoods**	**Region**	**No. of villages in WMA**	**Registered**
Savanna	Enduimet	High (GV+H)	Agro-pastoral	Arusha (Northern Tanzania)	9	2007
	Makame	Low (GV+H)		Manyara (Northern Tanzania)	4	2009
	Burunge	High (GV+H)		Manyara (Northern Tanzania)	10	2006
Miombo	Tunduru Nalika	Low (H)	Farm-based	Ruvuma (Southern Tanzania)	9	2003
	Mbarang'andu	Low (H)		Ruvuma (Southern Tanzania)	7	2006
	Liwale	Low (H)		Lindi (Southern Tanzania)	9	2003

*GV=game viewing; H=Hunting.

**Table 2:** Data sources for matching variables.

**Matching variable**	**Category**	**Year**	**Source**
Distance from major town	Market access	2002	Africover
Distance from major road	Market access	2002	Africover
Distance from wildlife corridor	Conservation	2008	Wildlife Corridors in Tanzania
Distance from protected area	Conservation	2013	World Database on Protected Areas
Total annual precipitation	Environmental	1997-2006 (mean)	Tropical Rainfall Measuring Mission
Elevation	Environmental	2000	Shuttle Radar Topography Mission
Slope	Environmental	2000	Shuttle Radar Topography Mission
Within elephant range	Conservation	2002	African Elephant Status Report 2002
Within lion range	Conservation	2008	IUCN Red List
Population density	Demographic	2002	Afripop
Proportion of forest	Environmental	2000	Global Land Cover 2000
Proportion of woodland and shrubs	Environmental	2000	Global Land Cover 2000
Proportion of grassland and crops	Environmental	2000	Global Land Cover 2000

**Table 3:** WMA study villages and matched non-WMA villages.

**Village**	**Site**	**District**	**North/ South**	**Matched to**
Olasiti and Kakoi (both part of Minjingu in 2007)	Burunge WMA	Babati	N	Gidemar
Magara, Manyara, Maweni (all part of Magara in 2007)		Babati	N	Kisangaji
*Mwada and Ngolei (both part of Mwada in 2007)		Babati	N	Namalulu
Sangaiwe		Babati	N	Magugu
Kitenden	Enduimet WMA	Longido	N	Selela
*Olmolog		Longido	N	Selela
Sinya		Longido	N	Oltukai
Tinga Tinga		Longido	N	Ngabobo
Irkiushoibor	Makame WMA	Kiteto	N	Kimotorok
Katikati		Kiteto	N	Gidemar
*Makame		Kiteto	N	Namalulu
Ndedo and Ngabolo (both part of Ndedo in 2007)		Kiteto	N	Kimotorok
*Barikiwa	Liwale WMA	Liwale	S	Mkutano
Kimambi		Liwale	S	Zinga Kibaoni
Mirui		Liwale	S	Kiperere
Mpigamiti		Liwale	S	Ngongowele
Kilimasera	Mbarang’andu WMA**	Namtumbo	S	Naikezi
Kitanda		Namtumbo	S	Mputa
Nambecha		Namtumbo	S	Mputa
Songaambele		Namtumbo	S	Chengena
Darajambili	Tunduru Nalika WMA **	Tunduru	S	Mangunguru
Kindamba		Tunduru	S	Mtengashari
Mbugalaji		Tunduru	S	Mangunguru
Ndenyende		Tunduru	S	Kitalo
*Selela*	Non-WMA villages	*Monduli*	N	Olmolog, Kitenden
*Oltukai*		*Monduli*	N	Sinya
*Kisangaji*		*Babati*	N	Magara, Manyara, Maweni
*Namalulu*		*Simanjiro*	N	Mwada and Ngolei, Makame
*Magugu*		*Babati*	N	Sangaiwe
*Gidemar*		*Babati*	N	Olasiti and Kakoi, Katikati
*Mputa*		*Namtumbo*	S	Kitanda, Nambecha
*Naikezi*		*Namtumbo*	S	Kilimasera
*Chengena*		*Namtumbo*	S	Songaambele
*Nangunguru*		*Tunduru*	S	Mbugalaji, Darajambili
*Mtengashari*		*Tunduru*	S	Kindamba
*Kitalo*		*Tunduru*	S	Ndenyende
*Mkutano*		*Liwale*	S	Barikiwa
*Kipelele*		*Liwale*	S	Mirui
*Zinga Kibaoni*		*Kilwa*	S	Kimambi
*Ngongowele*		*Liwale*	S	Mpigamiti
*Ngabobo*		*Meru*	N	Tinga Tinga
*Kimotorok*		*Simanjiro*	N	Irkiushoibor, Ndedo and Ngabolo

*=AA office is in this WMA village

**=AA office is not in a WMA village

**Table 4:** Household sampling strategy in each village.

	**Household head**	**Backup household head**	**Wife of male household head**	**Backup wife of household head**
Very poor	10	5	5	5
Leaders	10	5	5	5
Other	20	10	10	5
Total	40	20	20	15

**Table 5:** Structure of both household questionnaires.

**Section number**	**Questionnaire with household head**	**Questionnaire with wife of household head**
1	Introduction	Introduction
2	Household demographics	Household demographics
3	Overall well-being trends	Livestock
4	Shocks	Farm/garden
5	Land	Income generation
6	Livestock	Remittances
7	Bushmeat	Food security
8	Access to resources and mobility	*Participation in WMA decision-making
9	Environmental Income	Access to natural resources
10	Livelihood portfolio	Freedom of movement and safety
11	*Implementation of the WMA	External aid
12	Direct income and benefits	Ceremonies
13	Rule of law	*School sponsorship for children
14	Human casualties and injuries from wildlife and rangers/game scouts	*WMA benefits
15	Enumerator assessment of interviewee	*WMA costs
16		Enumerator assessment of interviewee
Sections that were only covered in WMA villages are indicated with *		

**Table 6:** Survey period and method.

**Dataset**	**Period of survey implementation**	**Recall (baseline)**	**Surveyed population (house-holds)**	**Survey method**	**Name of pdf file**	**Data file**
1; Wealth ranking	May 2014-March 2015	2007	13,578	Indirectly through focus group	PIMA_WEALTH_RANKING_CODE_LIST.pdf	PIMA_WEALTH_RANKING.csv
2; Household survey with household head	May 2014-May 2015	2007	1,924	Directly through questionnaire	PIMA_HHHEAD_SURVEY_INSTRUMENT.pdf	PIMA_HHHEAD_SURVEY.csv,
3; Household survey with wife of household head	May 2014-May 2015	2007	945		PIMA_WIFE_SURVEY_INSTRUMENT.pdf	PIMA_WIFE_SURVEY.csv,

**Table 7:** Summary of matching outcome for each of the variables used in the matching procedure.

**Matching variable**	**Tfm.**	**Before/After**	**WMA**	**Non-WMA**	**Std. Mean. Diff.**
Distance from major town	sqrt	Before	37.8	17.4	1.25
		After	39.7	37.5	0.12
Distance from major road	log	Before	11.6	7.0	0.44
		After	11.6	10.1	0.14
Distance from wildlife corridor	sqrt	Before	53.6	105.5	−0.95
		After	50.5	63.6	-0.24
Distance from protected area	sqrt	Before	22.8	43.5	−1.21
		After	25.3	33.5	−0.47
Total annual precipitation	log	Before	809.3	783.4	0.11
		After	733.3	746.8	−0.06
Elevation	sqrt	Before	881.8	833.8	0.11
		After	912.4	892.7	0.07
Slope	log	Before	2.2	3.3	−0.79
		After	1.9	2.0	−0.04
Within elephant range	none	Before	0.9	0.1	3.57
		After	0.9	0.9	0.00
Within lion range	none	Before	1.0	0.9	NA
		After	1.0	1.0	0.00
Population density	log	Before	34.2	1151.9	−18.54
		After	42.3	64.0	−0.27
Proportion of forest	none	Before	0.15	0.27	−0.47
		After	0.13	0.10	0.12
Proportion of woodland and shrubs	none	Before	0.42	0.35	0.19
		After	0.36	0.39	−0.08
Proportion of grassland and crops	none	Before	0.38	0.30	0.21
		After	0.48	0.50	−0.04
The column headed ‘Tfm.’ indicates the type of transformation applied to the variable prior to running the matching procedure to reduce the level of skew (sqrt=square root; ln=natural logarithm; none=no transformation applied). Mean values of matching variables within WMA and non-WMA villages included in the study, before and after matching are shown in the columns ‘WMA’ and ‘Non-WMA’. The standardised mean difference in the variable in WMA and non-WMA villages before and after matching is shown in the column ‘Std. Mean. Diff.’.					
